# Experimental Investigation of Aerodynamics of Feather-Covered Flapping Wing

**DOI:** 10.1155/2017/3019640

**Published:** 2017-12-21

**Authors:** Wenqing Yang, Bifeng Song

**Affiliations:** School of Aeronautics, Northwestern Polytechnical University, Xi'an 710072, China

## Abstract

Avian flight has an outstanding performance than the manmade flapping wing MAVs. Considering that the feather is light and strong, a new type of the flapping wing was designed and made, whose skeleton is carbon fiber rods and covered by goose feathers as the skin. Its aerodynamics is tested by experiments and can be compared with conventional artificial flapping wings made of carbon fiber rods as the skeleton and polyester membrane as the skin. The results showed that the feathered wing could generate more lift than the membrane wing in the same flapping kinematics because the feathered wing can have slots between feathers in an upstroke process, which can mainly reduce the negative lift. At the same time, the power consumption also decreased significantly, due to the decrease in the fluctuating range of the periodic lift curve, which reduced the offset consumption of lift. At the same time, the thrusts generated by the feather wing and the membrane wing are similar with each other, which increases with the increase of flapping frequency. In general, the aerodynamic performances of the feather wing are superior to that of the membrane wings.

## 1. Introduction

The flapping flight is thought to be potential to improve the low efficiency when the *Re* number is less than 10^5^. The natural flyers, birds, bats and insects, are very agile in different environments. The aim of manmade flapping wing MAVs (FMAVs) is to imitate the abilities or beauties of natural flyers as much as possible. The flight ability of manmade FMAVs is still far from their natural counterparts due to the current technology level of microelectronics technology, materials, and complex flow mechanism.

The aerodynamics of FMAVs is almost determined by the flapping wings, by its geometrics, kinematics, and by deforming manners. For the flapping flight, the flexible deformation has an important effect on the thrust generation. There are a few net thrusts generated for a rigid flapping movement; the thrust generated by rigid wings is far less than that of flexible wings. The aerodynamics and flexible deformation are tightly connected to each other; their relation is a key to design an efficient flapping wing [1–4].

Flexible deformation can be divided into two categories. One kind deforms passively, like most FMAVs, like the Nano Hummingbird [5], the BionicOpter [6], and so on [7–10]. The other kind can deform actively in a certain extent, such as the Smartbird [11]. Even for the active deformation, it is not completely active; there are still some passive deformations happening, for example, the outer part of wings or trailing edges.

For the insects, wing kinematics is determined totally by the muscle of the wing root. That is similar to most FMAVs; the wing is driven on the root by the flapping mechanism. All the deformation of the wing is passive. A lot of investigations have been published about the wings of insects. They showed apparent deformation in chord- and span-wises [12–15].

For the birds, the wing can do some complex motions due to the skeletal structures like human arms, such as torsion, bending, or folding. That is to say, the input movement of the bird wings is more complicated than that of the insect wings. However, if you look at the outer part and the rear part of the wings, which are called the primaries and secondaries [16], the deforming manners of these feathers are also passive deformation as only the root is driven.

In general, birds and insects fly in a partly similar way; the difference is the input of movement manners. That is to say, passive deformation mostly decides the aerodynamics. If we want to have an outstanding FMAV, the flexible structure and kinematics should be designed carefully.

The wings of birds are covered with feathers, which must have a very important effect on the high efficiency. The muscle of the bird wing is hard to imitate or use. The feathers are relatively easy to be obtained, such as the wing feathers of goose or duck. At present, the carbon fiber rods and polyester film are a common selection for making a flapping wing, for their light mass, good elastics, and high strength. Comparing the feather with the carbon fiber rod, we can find that the feather has a perfect structure. The feather is made of keratin, which is elastic, light, and strong. It is a cylindrical tube in cross section at their base.

Here comes out the research idea: whether a FMAV will have a better performance or not when the wings are covered with feathers. Hence, the feathered flapping wings are made and tested and compared with a polyester membrane wing.

## 2. Experimental Setup

### 2.1. Flapping Mechanism

**Figure 1:**
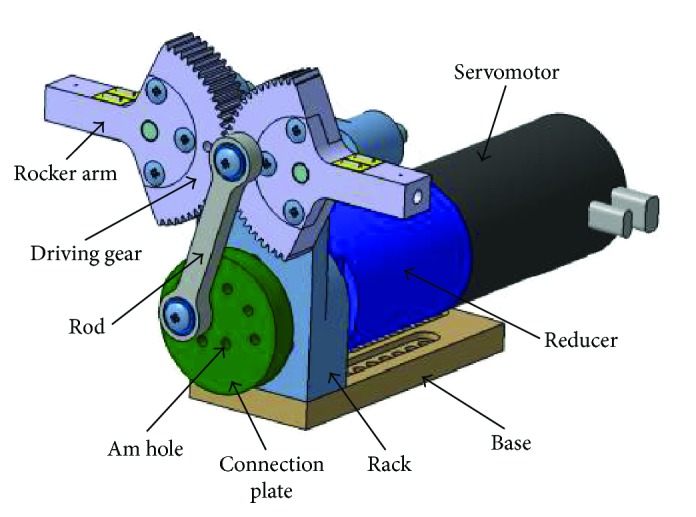
The flapping mechanism.

The flapping mechanism is composed of the servomotor, reducer, rack, rod, gear, rocker arm, connecting plate, and installation frame, with maximum horizontal and vertical dimensions of 53 mm and 62 mm, respectively, as shown in Figure 1.

As the direct output of power components, the performance of the driving motor is often directly determining the performance of a complete set of flapping mechanism. The German Faulhaber series of 2057s servomotor is chosen as the driving motor.

The motor adopts a double-pole design. The usage of messenger, hollow glass, tilt winding, and rotor winding makes it get a high power level and high dynamic performance within the scope of a small size and lightweight. The motor can realize complicated digital communication and control through the CAN bus. The internal control mode can control the speed and position precisely and quickly because of closed-loop control.

Due to a variety of sensors that are integrated internally, the motor can return the real-time position, speed, current, voltage, and other parameters in the process of running. At the same time, the motor has a planetary gear reducer without return difference with a reduction ratio of 43 : 1. The output speed is consistent with the flapping frequency; therefore, there is no need to design an additional gear reducer.

The flapping mechanism adopts four connecting rod designs. The motor drives the output plate, which connects the rod to make the rocker flapping around the gear transmission. There are several holes to control the flapping amplitude; each am-hole has a different distance to the center of the output plate, and a different am-hole actually represents a different crank length.

### 2.2. Nano17 Force and Torque Sensor

The aerodynamic force generated by the flapping wing is small in absolute value and periodic alternating that has a special requirement to the force measurement. Ordinary six-component balance always has difficulties achieving small range measurement and has a bad dynamic response. For example, the cassette balance has big size, which will have interference on aerodynamic performance; the lever balance has small stiffness, which will probably induce vibration under the cyclical load that is not conducive to measure the flapping wing.

The Nano17 SI-12-0.12 force and torque sensor of ATI Company are chosen as the balance of flapping wing experiment. These series sensors have a micro hub-spoke design, with high strength stainless steel or titanium alloy as strain components, and adopt the high sensitivity silicon strain gauge. It is suitable for micro flapping wing wind tunnel experiment for its relatively large range, small volume, high sensitivity, fast response speed, and big stiffness. The balance has been applied to several micro flapping wing tests by several research teams. Some key parameters of the sensor are shown in Table 1.

### 2.3. Flexible Flapping Wing

**Figure 2:**
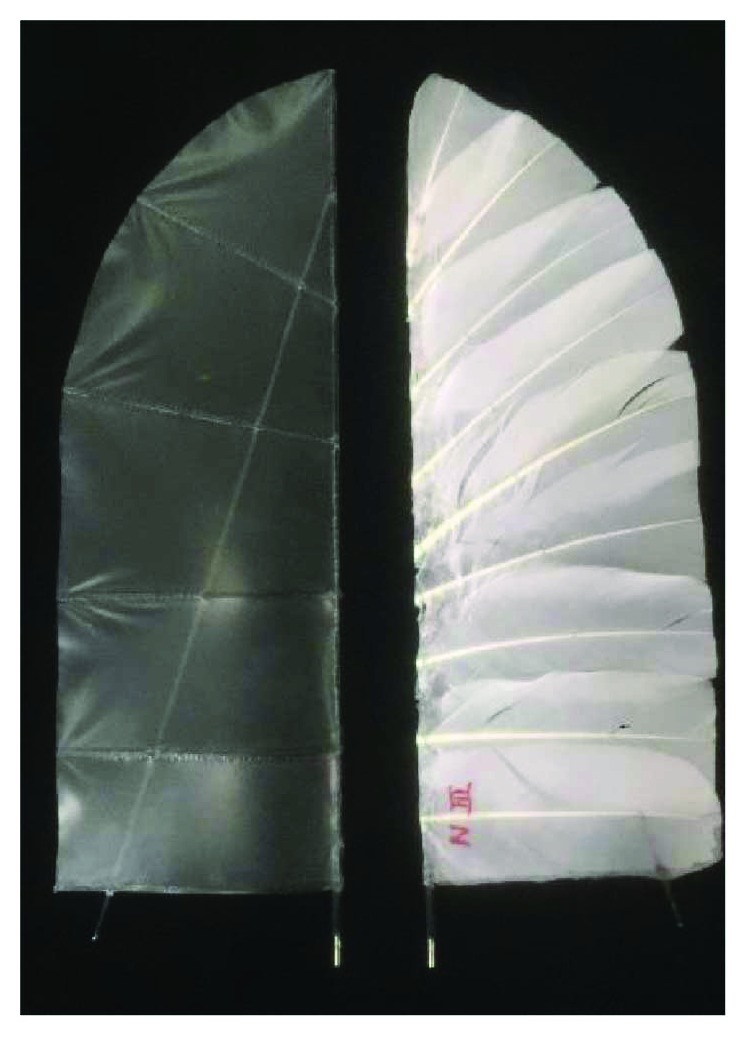
Membrane wing (left) and feather wing (right).

We test two kinds of flapping wings. One is made of carbon fiber and polyester membrane, as shown in Figure 2 left. This wing is a basal model, which is also our daily use structure. The other kind of flapping wing has a feather cover, as shown in Figure 2 right, which has the same shape and area with the basal wing for the comparison. The two kinds of wings have the same size, with a half span of 26 cm and root chord of 10 cm. The main structure skeleton is also the same which is composed of a leading spar and a slant spar. The membrane wing has a mass of 6.9 g, and the feather wing has a mass of 7.1 g; they are similar in mass. The order of the feathers on the flapping wing is like the arrangement of the bird wing. In the two adjacent feathers, the one near the wing tip is below, and the one near the wing root is above.

## 3. Results and Discussion

The aerodynamic performance and corresponding power of the two kinds of flapping wings are tested at different frequencies in the absence of wind. This state corresponds to the actual situation that can be considered a hovering flight status, belonging to a special state of the flapping wing flight envelope. The purpose of the experiment was to test their basic performance as a comparison. Each experiment was recorded three times, and the average of the three data was analyzed. The flapping amplitude is 61.0 degree from top to bottom.

**Figure 3:**
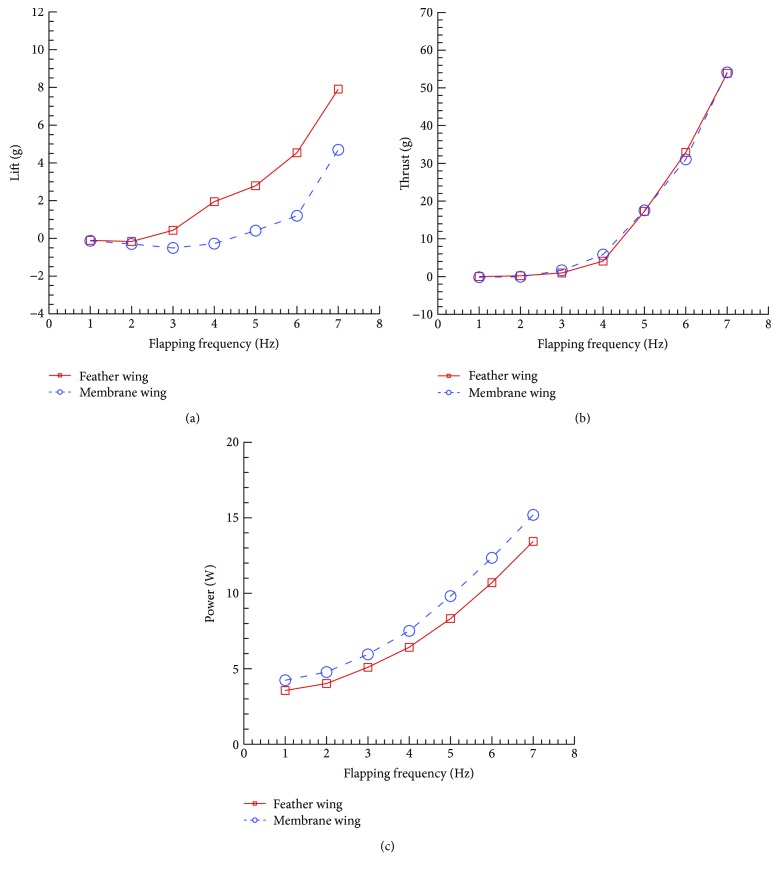
The averaged forces and power comparison between the feather wing and the membrane wing.

The averaged lift force, thrust force, and consumed power of the two wings which vary with the flapping frequency are shown in Figure 3.

The lift and thrust are two components of the measured forces. The model is installed horizontally. According to the traditional definition, the force in the vertical direction is called lift, and the force in the horizontal direction is called thrust. In fact, the net force of both is the vector force produced by the flapping wing. In this windless situation, there is no aerodynamic lift caused by the flow, so the measurement forces are totally generated by the flapping wings. An air vehicle can hover when the net force is greater than gravity.

The power used in the experiment is the electrical power, which is given by the current times the voltage. The power is actually consumed by the whole system, including the mechanism and the motor. Considering that the power consumption of the mechanism and the motor is also related to the wing load and the mechanism and the motor are also part of the experimental system, the total power is used to analyze finally.

Due to the camber in chord-wise of the two wings, both flapping wings produced pure positive lift in the case of symmetric flapping motion. However, the feather wing produced more lift than the membrane wing especially when the flapping frequency is bigger than 4 Hz shown in Figure 3(a). The thrusts generated by the two wings are almost the same, which increase with the increase of flapping frequency. At the same time, the consumed power of the feather wing is less than that of the membrane wing.

**Figure 4:**
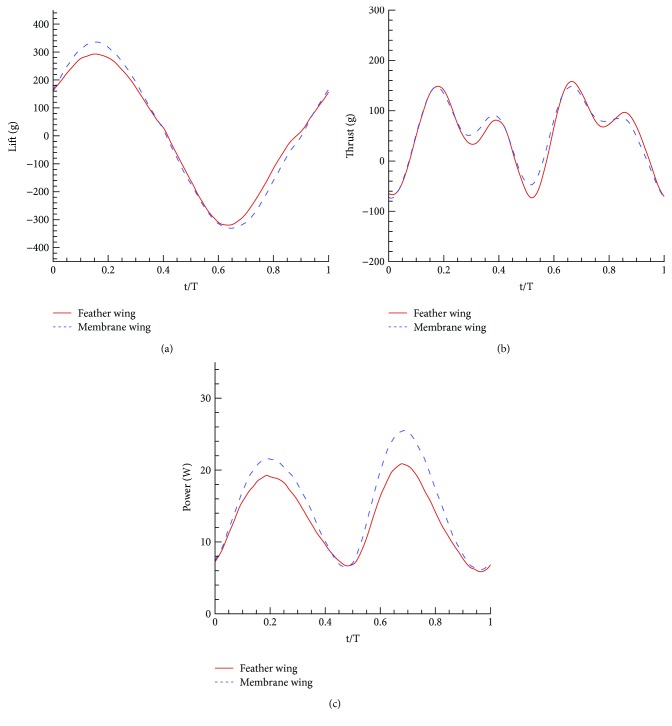
The periodical forces and power comparison between the feather wing and the membrane wing.

The periodic curves of lift, thrust, and consumed power in a flapping cycle at 7 Hz frequency are shown in Figure 4. The sample rate of the experimental system is 2000 points per second, so the results appear to be continuous.

For the cycle lift curve, the lift force of the feather wing is less than that of the membrane wing during downstroke process, but opposite in the upstroke process. In the whole flapping period, the amount of increase is greater than that of the decrease, so the total lift increases for the feather wing. The fluctuating range of the feather wing is small than that of the membrane wing.

For the thrust curve, the thrust of the feather wing is less than that of the membrane wing during downstroke process, but opposite in the upstroke process. However, in the whole period, the amount of increase and decrease is almost the same.

For the consumed power curve, the power of the feather wing is less than that of the membrane wing during the whole flapping cycle; that is mainly because the lift fluctuating range of the feather wing is small to reduce the power consumption.

**Figure 5:**
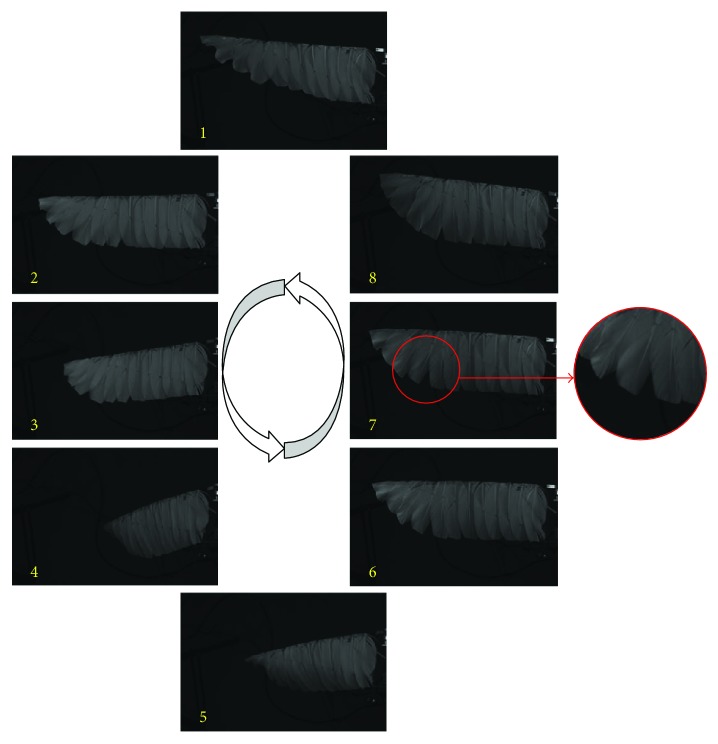
Flexible deformation of the feather wing in a whole flapping cycle.

The whole process was filmed with a high-speed camera. For both wings, the apparent deformation can be observed in the whole flapping process. The maximum deformation and the amount of time occur similarly. The main difference between the two wings is happening in the upstroke; there is a noticeable gap happening between adjacent feathers of feather wings shown in Figure 5. This behavior significantly reduces the negative lift from the upstroke process; this is also the secret of the feather wing to generate more lift.

In the photos, the trailing edge deformation of the feather wing is a little greater than that of the membrane wing, because the trailing edge of the feather is very thin and its stiffness changes continuous. This feature makes the range of lift fluctuate less, which is one of the things that man-made materials cannot do. It is the decrease in the lift amplitude that leads to the reduction of consumed power.

Considering the effect of inertia force on power, the power consumption of the wing skeletons is measured. We made wing skeletons of the above two flapping wings for testing. The skeleton of the feather wing is obtained by cutting both sides of each feather, retaining only the middle shaft of every feather.

**Figure 6:**
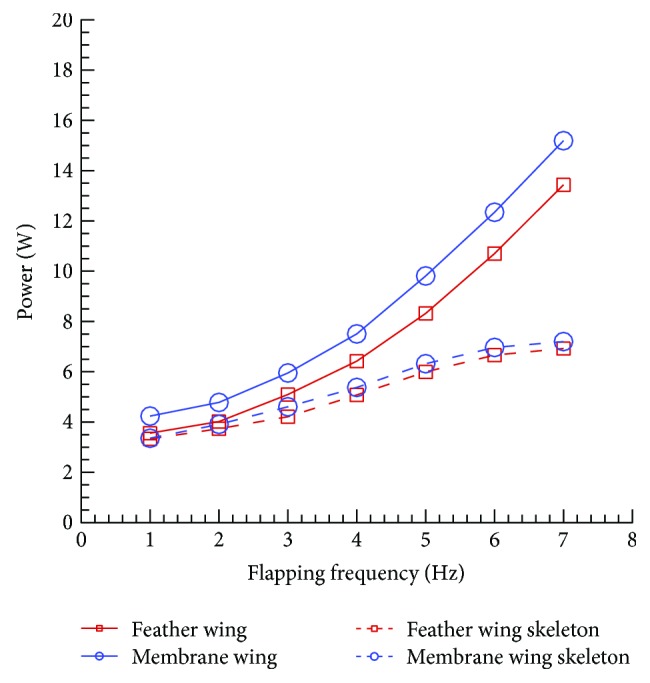
The power consumption of wing skeletons, considered as the power consumption of inertial forces.

The aerodynamic force of the pure skeleton is very small and can be ignored. The energy consumed by the skeleton flapping is regarded as the energy consumed by the inertial force. The power consumption of the wing skeleton at different frequencies is shown in Figure 6. As the frequency increases, the proportion of the inertial force consumption decreases gradually. The reason might be as the frequency increases, the aerodynamic forces become larger and dominant. The power consumption of both wing skeletons is close to each other. Because the inertial force consumed much power, the flapping wing should be made of lightweight and high strength material to reduce the inertial force consumption.

**Figure 7:**
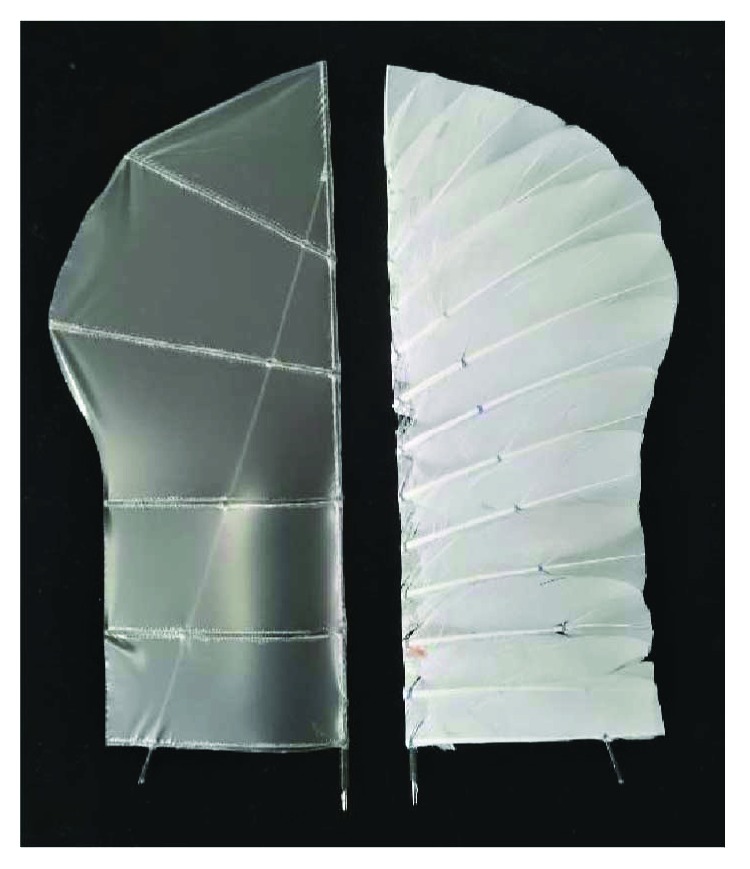
Another layout of flapping wing for verification.

Due to the limitations of the study of one layout of wings, we have also designed and produced a different layout of the flapping wing for comparison, as shown in Figure 7. Unlike the wings described above, this new layout increases the outer area of the trailing edge, which can produce a greater thrust in flapping motion. These two layouts have the same root chord length and span length. The wings of this layout also have approximate weight, and the mass of a feather wing is 7.9 g and a membrane wing is 7.7 g.

**Figure 8:**
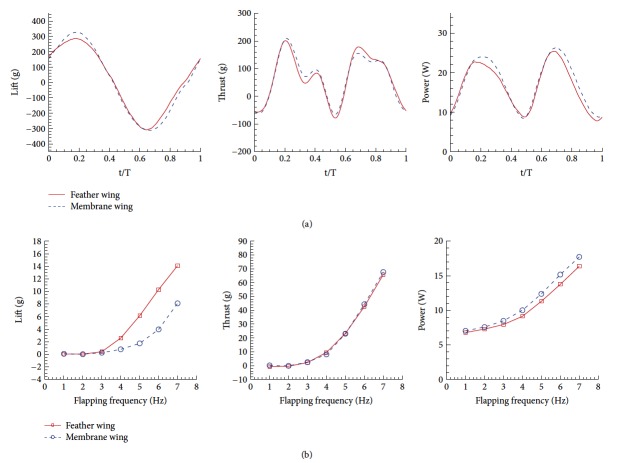
The forces and power comparison between the feather wing and the membrane wing of the other layout: (a) periodical results and (b) averaged results.

In the same experimental parameters, the test results of the new layout are shown in Figure 8. It can be seen that the results are similar to the former layout, which is the aerodynamic performance of the feather flapping wing which is better than that of the membrane flapping-wing. During the experiment, it can also be observed that the feather flapping wing will have the effect of the gap between feathers during the upstroke process, similar to the phenomenon in Figure 5.

The results of this study are in accordance with the “venetian blind effect” [17, 18] of natural birds in a certain extent. It illustrates the wonders of nature. The outstanding performance of feathers deserves further study, and the feathers are suitable for improving the performance of artificial MAV.

Since we did not obtain the optimal aerodynamic layout of the two types of flapping wings, this paper does not study the optimal feather wing and the optimal membrane wing. Because of the different mechanisms between the two kinds of flapping wings, perhaps, the optimal feather wing layout is not consistent with the optimal membrane wing layout. However, through the study of this paper, to some extent, it can be explained that under the same layout, the feather wing has better aerodynamic performance and power characteristic than the membrane wing.

## 4. Conclusions

The aerodynamics of a flapping wing covered by feathers is designed and tested experimentally. The order of the feathers on the flapping wing is like the arrangement of the birds' wing. In the two adjacent feathers, the one near the wing tip is below, and the one near the wing root is above. Because the feathers are independent of each other, there will be gaps between the two adjacent feathers when the aerodynamic pressure reaches a certain point.

In the case of the wings flapping around the wing root, the area near the wing tip has larger range movement and accordingly larger aerodynamic pressure than the area near the wing root, which causes larger deformation near the wing tip.

In the downstroke process, the arrangement manner makes the wing as a whole surface. However, in the upstroke process, there will be gaps appearing between feathers, which let some air passed and accordingly decrease the negative lift generated during upstroke, which results in the averaged lift increase in a whole flapping cycle. At the same time, the fluctuating range of the lift curve in a cycle decreases, which also decreases the consumed power.

According to the results of experiments, the thrusts generated by the feathered wing and membrane wing are almost identical with each other. And they increase as the flapping frequency increases. This phenomenon indicates that the thrust is mainly related to the flapping frequency.

In contrast to the situation of the same thrust, the feather wing can produce a greater lift and consume less power; it means the aircraft with feather wings can fly more easily and has a longer endurance. Because of the greater lift, the angle of attack can be reduced in cruise stage, and the drag is reduced accordingly; the power consumption is reduced further.

The feather wing also has a disadvantage. Because the feathers are not from the pair of wings of the same goose but are bought from the market, they have poor symmetry. To make a pair of wings, it is needed to be carefully picked, as there are barely very same two feathers. At the same time, the process of manual production also brings a certain amount of error.

The results of this paper show that the natural feather is superior to the manmade membrane. The feathers of poultry are easy to obtain and cheap to use to improve the performance of FMAV. It also improves the appearance of FMAV and looks more like a bird flying in the air.

## Figures and Tables

**Table 1:** Parameters of Nano17 sensor.

Force and torque sensor	Component	Range	Resolution	Stiffness
Nano17 SI-12-0.12 Φ17 × 15 mm	Fx	12 N	1/320 N	8.2 × 106 N/m
	Fy	12 N	1/320 N	8.2 × 106 N/m
	Fz	17 N	1/320 N	11 × 106 N/m
	Tx	120 Nmm	1/64 Nmm	240 Nm/rad
	Ty	120 Nmm	1/64 Nmm	240 Nm/rad
	Tz	120 Nmm	1/64 Nmm	380 Nm/rad

## References

[1] Shyy W., Berg M., Ljungqvist D. (1999). Flapping and flexible wings for biological and micro air vehicles. *Progress in Aerospace Sciences*.

[2] Tanaka H., Okada H., Shimasue Y., Liu H. (2015). Flexible flapping wings with self-organized microwrinkles. *Bioinspiration & Biomimetics*.

[3] Heathcote S., Wang Z., Gursul I. (2008). Effect of spanwise flexibility on flapping wing propulsion. *Journal of Fluids and Structures*.

[4] Liu P., Bose N. (1997). Propulsive performance from oscillating propulsors with spanwise flexibility. *Proceedings of the Royal Society A: Mathematical, Physical and Engineering Sciences*.

[5] Keennon M., Klingebiel K., Won H., Andriukov A. Development of the nano hummingbird: a tail-less flapping wing micro air vehicle.

[6] Festo.com [Internet] http://www.festo.com/group/en/cms/10224.htm.

[7] Yang L., Esakki B., Chandrasekhar U., Hung K., Cheng C. (2015). Practical flapping mechanisms for 20 cm-span micro air vehicles. *International Journal of Micro Air Vehicles*.

[8] Phan H. V., Kang T., Park H. C. (2017). Design and stable flight of a 21 g insect-like tailless flapping wing micro air vehicle with angular rates feedback control. *Bioinspiration & Biomimetics*.

[9] de Croon G. C. H. E., de Clercq K. M. E., Ruijsink R., Remes B., de Wagter C. (2009). Design, aerodynamics, and vision-based control of the DelFly. *International Journal of Micro Air Vehicles*.

[10] Gerdes J. W., Gupta S. K., Wilkerson S. A. (2012). A review of bird-inspired flapping wing miniature air vehicle designs. *Journal of Mechanisms and Robotics*.

[11] Festo.com [Internet] https://www.festo.com/group/en/cms/10238.htm.

[12] Sane S. P. (2003). The aerodynamics of insect flight. *Journal of Experimental Biology*.

[13] Walker S. M., Thomas A. L. R., Taylor G. K. (2010). Deformable wing kinematics in free-flying hoverflies. *Journal of the Royal Society Interface*.

[14] Zhao L., Huang Q., Deng X., Sane S. P. (2010). Aerodynamic effects of flexibility in flapping wings. *Journal of the Royal Society Interface*.

[15] Wootton R. (1981). Support and deformability in insect wings. *Journal of Zoology*.

[16] Pennycuick C. J. (2008). *Modelling the Flying Bird*.

[17] Tobalske B. W. (2000). Biomechanics and physiology of gait selection in flying birds. *Physiological and Biochemical Zoology*.

[18] Norberg U. M., Wu T. Y., Brokaw C. J., Brennen C. (1975). Hovering flight in the pied flycatcher (Ficedula hypoleuca). *Swimming and Flying in Nature*.

